# Author Correction: A randomized controlled trial of alpha phase-locked auditory stimulation to treat symptoms of sleep onset insomnia

**DOI:** 10.1038/s41598-024-72802-4

**Published:** 2024-09-19

**Authors:** Scott Bressler, Ryan Neely, Ryan M. Yost, David Wang

**Affiliations:** 1 Elemind Technologies, Inc., Cambridge, MA USA; 2 Science and Research, Elemind Technologies, Inc., Cambridge, MA 02139 USA

Correction to: *Scientific Reports*10.1038/s41598-024-63385-1, published online 06 June 2024

The original version of this Article contained an error in Figure 5b, where the labels in the y-axis were not aligned.

**Figure 5:**
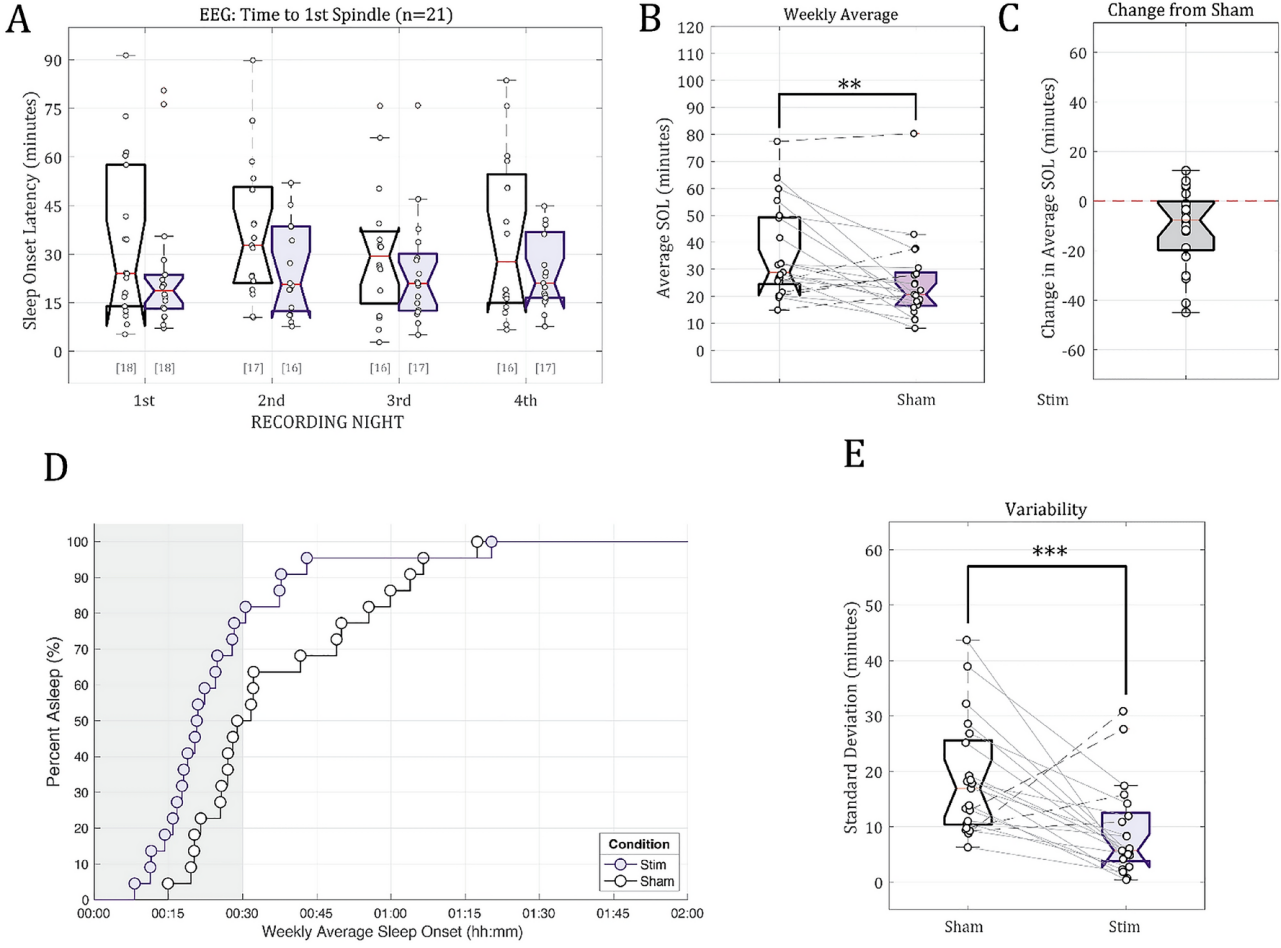
Sleep onset as defined by the time (in minutes) of the first visually-identified spindle in the EEG data. (**A**) Individual subject time to 1st spindle estimates for the Sham control condition (white) and the phase-locked auditory stimulation condition (purple). The bracketed numbers below show the number of valid data logs (out of 21 subjects). (**B**) The across-night weekly average latency to 1st spindle. Individual subject trends across conditions are plotted using connecting lines between data points. Subjects whose average sleep onset latency decreased are connected with solid lines; subjects whose average weekly sleep onset times increased are connected with dashed lines. (**C**) Per-subject difference from Sham (no stimulation) weekly sleep onset latency estimates due to phase-locked stimulation (Stim—Sham). Subjects with faster weekly sleep onset times have negative values. (**D**) Survivor plot showing the weekly average time to fall asleep for all participants in the Stim (purple circles) or Sham (white circles) conditions. The shaded gray bar represents the 30-min stimulation period (Stim sessions only). (**E**) Weekly standard deviation in subjects’ sleep onset times (in minutes). Standard deviations in sleep onset times were calculated from available data from the four nights of recorded EEG data for each of the two conditions ** indicates p < 0.05, *** indicates p < 0.001.

The original article has been corrected.

